# Evaluation of autoantibodies to desmoglein-2 in dogs with and without cardiac disease

**DOI:** 10.1038/s41598-023-32081-x

**Published:** 2023-03-28

**Authors:** Ashley L. Walker, Ronald H. L. Li, Nghi Nguyen, Carina E. Jauregui, Kathryn M. Meurs, Allison L. Gagnon, Joshua A. Stern

**Affiliations:** 1 William R. Pritchard Veterinary Medical Teaching Hospital, University of California, Davis, CA USA; 2 Department of Surgical and Radiological Sciences, School of Veterinary Medicine, University of California, Davis, CA USA; 3 Department of Medicine and Epidemiology, School of Veterinary Medicine, University of California-Davis, 2108 Tupper Hall, Davis, CA 95616-8732 USA; 4 College of Veterinary Medicine, North Carolina State University, Raleigh, NC 27604 USA

**Keywords:** Genotype, Mutation, Biomarkers, Health care, Medical research, Cardiology, Cardiovascular diseases, Arrhythmias, Cardiomyopathies

## Abstract

Autoantibodies to desmoglein-2 have been associated with arrhythmogenic right ventricular cardiomyopathy (ARVC) in people. ARVC is a common disease in the Boxer dog. The role of anti-desmoglein-2 antibodies in Boxers with ARVC and correlation with disease status or severity is unknown. This prospective study is the first to evaluate dogs of various breeds and cardiac disease state for anti-desmoglein-2 antibodies. The sera of 46 dogs (10 ARVC Boxers, 9 healthy Boxers, 10 Doberman Pinschers with dilated cardiomyopathy, 10 dogs with myxomatous mitral valve disease, and 7 healthy non-Boxer dogs) were assessed for antibody presence and concentration via Western blotting and densitometry. Anti-desmoglein-2 antibodies were detected in all dogs. Autoantibody expression did not differ between study groups and there was no correlation with age or body weight. In dogs with cardiac disease, there was weak correlation with left ventricular dilation (r = 0.423, p = 0.020) but not left atrial size (r = 0.160, p = 0.407). In ARVC Boxers there was strong correlation with the complexity of ventricular arrhythmias (r = 0.841, p = 0.007) but not total number of ectopic beats (r = 0.383, p = 0.313). Anti-desmoglein-2 antibodies were not disease specific in the studied population of dogs. Correlation with some measures of disease severity requires further study with larger populations.

## Introduction

Arrhythmogenic right ventricular cardiomyopathy (ARVC) is an inherited myocardial disease of the Boxer dog that results in ventricular arrhythmias, syncope, increased risk of sudden death, and in some cases, ventricular dilation and systolic dysfunction^1–3^. The pathophysiology and histopathological changes in ARVC are similar in the Boxer and in people^4–6^. While the specific mechanisms leading to ARVC are not completely understood, dysfunction of desmosomal proteins at the level of the intercalated disc may play a role in destabilization of structural integrity and disruption of the electrical conduction of the myocardium^7,8^. An autosomal dominant mutation in the gene encoding striatin, a protein that co-localizes to the intercalated disc and is associated with desmosomal proteins, has been associated with development of the disease with incomplete penetrance in North American Boxer dogs^9^. Not all dogs with the mutation go on to develop clinical disease, but there is evidence that homozygous mutants can develop a more severe phenotype^3,10^. Additionally, Boxers negative for the striatin mutation can develop clinically significant ARVC. Thus, genetic testing alone does not allow accurate discrimination between healthy and affected Boxers. In humans, more than a dozen mutations in various desmosomal proteins and a smaller number of non-desmosomal genes have been shown to play a role in the development of ARVC^11^. Despite this, in approximately 50% of human ARVC cases a genetic mutation is not identified^12,13^. Therefore, diagnosis of both human and canine ARVC remains challenging. Currently, in Boxer dogs there is no universally accepted criteria to diagnose ARVC, but a diagnosis is typically made based on the number and complexity of ventricular ectopy (VE) on 24-h ambulatory ECG (AECG) without another etiology for the arrhythmias or in the case of a dilated and poorly contractile left ventricle without other etiology^2,5^.

More recently, there has been investigation into a potential autoimmune component of ARVC. Two studies have evaluated for the presence of autoantibodies in humans with ARVC, with one of these also evaluating a small cohort of Boxer dogs^14,15^. In the first study, by Chatterjee et al., autoantibodies to the desmosomal protein, desmoglein-2 were found in almost all human patients with ARVC and were absent in almost all healthy subjects and patients with hypertrophic cardiomyopathy or dilated cardiomyopathy (DCM)^14^. Furthermore, anti-desmoglein-2 antibodies were identified in 10/10 of the Boxer dogs with ARVC and absent in their control dog cohort. The burden of anti-desmoglein-2 antibodies correlated positively with the number of VE beats and were present in patients without an identified ARVC genetic mutation in the human cohort studied. Evaluation of whether antibody density correlated with disease severity in the Boxer cohort was not conducted. Therefore, whether autoantibody presence could serve as a prognostic indicator in addition to having diagnostic potential in the Boxer remains unknown. A second study by Caforio et al. evaluated a larger population of humans with ARVC, affected relatives, and healthy relatives for the presence of anti-heart or anti-intercalated disc antibodies^15^. While the incidence of anti-heart and anti-intercalated disc antibodies was higher in patients with ARVC and affected relatives, autoantibodies were also found in healthy relatives, people with other non-inflammatory cardiac disease, and healthy blood donors at a lower frequency^16^.

There is a lack of research assessing the role of autoimmunity in other common canine cardiac diseases, with only a few studies to date. Autoantibodies to the adrenergic, beta-1 receptor have been documented in both humans and Doberman Pinschers with DCM^17–20^. However, these are considered “functional antibodies” that bind to G protein coupled receptors, possibly inducing disturbed metabolic balance or stimulating pathologic conditions^21^. Classical anti-cardiac antibodies that result in tissue destruction and immune activation have not been studied in canine DCM. No studies to date have evaluated any anti-cardiac antibodies in dogs afflicted with the most common canine cardiac disease, myxomatous mitral valve disease (MMVD).

The specific aims of this study were to determine if autoantibodies against the desmosomal protein desmoglein-2 are present in Boxer dogs with ARVC and whether they are disease and/or breed specific by also evaluating healthy Boxer dogs, Doberman Pinschers with DCM, small breed dogs with MMVD, and healthy non-Boxer dogs. A secondary aim was to determine whether the expression of these autoantibodies correlates with arrhythmia severity found on AECG and/or the degree of structural disease on echocardiography. We hypothesized that autoantibody expression would segregate dogs with cardiac disease from healthy dogs but not be disease specific. We also hypothesized that autoantibody expression would correlate with arrhythmia burden and the degree of structural cardiac disease.

## Results

### Study populations

Of the 46 dogs enrolled into the study, there were 10 dogs each in the ARVC Boxer, DCM Doberman Pinscher, and MMVD small breed groups; 9 were healthy Boxer (HB) dogs, and 7 were healthy non-Boxer dogs (C group). Demographic and clinical characteristics for each study dog are shown in Table 1. As anticipated due to known breed and disease associations, body weight, age, and globulin concentrations significantly differed between some of the groups. Specifically, body weight differed between the MMVD group and all other groups (ARVC p < 0.0001; DCM p < 0.0001; HB p < 0.0001; healthy non-Boxers p = 0.0049), as well as, between the HB and healthy non-Boxer group (p = 0.0219) and the DCM and healthy non-Boxer groups (p = 0.0002). Age differed significantly between the MMVD and healthy non-Boxer groups (p < 0.0001), HB group (p < 0.0001), and the ARVC group (p = 0.0108). Age also significantly differed between the DCM and HB groups (p = 0.0296) and the DCM and healthy non-Boxer group (p = 0.044). Age did not differ between the ARVC and HB groups (p = 0.1475). The only significant difference in globulin concentrations was between the MMVD and the healthy non-Boxer groups (p = 0.0074).

**Table 1:** Study population characteristics including body weight (kilograms), age (years), globulin (g/dL), sex, relevant mutation status, ARVC type, and ACVIM MMVD stage.

Dog number	Body weight (kg)	Age (yrs)	Globulin (g/dL)	Sex	Striatin mutation	ARVC type
*ARVC-1*	35.4	2	2.5	MC	Heterozygous	II
*ARVC-2*	25	9	2.8	MC	Heterozygous	II
*ARVC-3*	36.8	10	2.7	FS	Heterozygous	II
*ARVC-4*	22	4	2.3	FS	Heterozygous	II
*ARVC-5*	40.6	10	2.3	FS	Heterozygous	II
*ARVC-6*	26.6	1	2.2	FI	Heterozygous	II
*ARVC-7*	21.8	10	2.1	FS	Heterozygous	II
*ARVC-8*	32.9	2	1.9	MI	Homozygous	III
*ARVC-9*	24.6	11	3.3	FS	Homozygous	II
*ARVC-10*	21.4	9	2.5	FS	Homozygous	III
*ARVC overall*	28.7 (± 7.1)	6.8 (± 4)	2.5 (± 0.4)			

Dog number	Body weight (kg)	Age (yrs)	Globulin (g/dL)	Sex	Striatin mutation	
*HB-1*	32.2	3	2.7	MI	Negative	
*HB-2*	24.5	3	2.6	FI	Negative	
*HB-3*	26	3	2.0	FI	Negative	
*HB-4*	41	6	2.3	MI	Negative	
*HB-5*	31	1	2.1	MI	Negative	
*HB-6*	36.2	6	2.7	MI	Negative	
*HB-7*	29.8	5	2.3	FI	Negative	
*HB-8*	22.8	5	2.6	MI	Negative	
*HB-9*	31	2	2.0	MI	Negative	
*HB overall*	30.5 (± 5.7)	3.8 (± 1.8)	2.4 (± 0.3)			

Dog number	Body weight (kg)	Age (yrs)	Globulin (g/dL)	Sex	DCM1 mutation	DCM2 mutation
*D-1*	39	6	2.6	MI	Homozygous	Homozygous
*D-2*	37.8	8	2.3	MI	Heterozygous	Heterozygous
*D-3*	58.3	7	2.5	MC	Heterozygous	Negative
*D-4*	29.5	8	2.4	FS	Negative	Negative
*D-5*	35.4	7	2.6	MI	Heterozygous	Negative
*D-6*	29.5	12	2.7	MC	Heterozygous	Homozygous
*D-7*	34.3	8	2.1	FS	Heterozygous	Heterozygous
*D-8*	32.6	9	1.9	FS	Heterozygous	Heterozygous
*D-9*	28.2	8	3.1	FI	Negative	Homozygous
*D-10*	34	4	2.5	MI	Negative	Heterozygous
*D overall*	35.9 (± 8.7)	7.7 (± 2.1)	2.5 (± 0.3)			

Dog Number	Body weight (kg)	Age (yrs)	Globulin (g/dL)	Sex	ACVIM stage	
*M-1*	9.2	12	2.5	MC	B2	
*M-2*	7.6	7	3.3	MC	C	
*M-3*	5.8	14	3.4	FS	C	
*M-4*	7.3	7	2.0	FS	B2	
*M-5*	6	14	3.2	FS	B2	
*M-6*	7.1	12	2.5	MC	C	
*M-7*	11.4	8	2.1	MC	C	
*M-8*	7.3	15	2.7	MI	C	
*M-9*	4.3	11	2.3	FS	B2	
*M-10*	4.8	11	2.5	FS	B2	
*M overall*	7.1 (± 2.1)	11.1 (± 2.9)	2.7 (± 0.5)			

Dog number	Body weight (kg)	Age (yrs)	Globulin (g/dL)	Sex	Breed	
*C-1*	13.6	8	1.9	FS	Dachshund cross	
*C-2*	32.3	4	1.9	MC	Labrador retriever	
*C-3*	15.7	5	2.7	FS	Taiwanese dog	
*C-4*	5.7	3	1.7	MC	Terrier cross	
*C-5*	21.5	1	1.6	FS	Golden retriever	
*C-6*	27.2	2	1.8	FS	Alaskan husky	
*C-7*	21.1	3	2.2	FS	Border collie	
*C overall*	20.9 (± 9)	4.1 (± 2.4)	2.0 (± 0.4)			

Dogs are listed by group: Boxers with ARVC (ARVC−), healthy boxers (HB−), Doberman Pinschers with DCM (D−), small breed dogs with MMVD (M−), and healthy non-Boxer dogs (C−). Mean (+/− SD) are reported for continuous variables for each group.

Nine out of the ten Boxers with ARVC had at least one 24-h AECG performed. The single Boxer (ARVC-9) without an ambulatory ECG (AECG) was presented to the clinic in sustained ventricular tachycardia refractory to medical management and was subsequently hospitalized and humanely euthanized. Results of the nine Boxers with AECG performed are shown in Table 2 alongside relevant anti-arrhythmic medications and the results of prior AECG at the time of ARVC diagnosis, if performed.

**Table 2:** Results of AECG recordings in the nine ARVC Boxers that had at least one ECG performed.

Dog number	Total VE on AECG	VE complexity	Medications during AECG	Total VE prior EACG	VE complexity prior AECG	Medications during prior AECG
*ARVC-1*	2471	4	–	–	–	–
*ARVC-2*	2265	4	–	–	–	–
*ARVC-3*	33,056	3	Sotalol	–	–	–
*ARVC-4*	147	1	Sotalol, mexiletine	1714	1	Sotalol
*ARVC-5*	223	1	Sotalol	8879	3	Sotalol
*ARVC-6*	932	3	–	–	–	–
*ARVC-7*	74	2	Sotalol	–	–	–
*ARVC-8*	20	1	–	–	–	–
*ARVC-10*	1198	4	Sotalol	–	–	–

Results of the recording more recently performed prior to sample collection and a previous AECG at diagnosis of ARVC are displayed, including the total number of ventricular ectopies (VE) and the complexity of ectopy along with relevant anti-arrhythmic medication being administered at the time of recording.

All healthy Boxers had an unremarkable echocardiogram and 5-min surface ECG, with no ectopy observed. On 24-h AECG all nine healthy Boxers had rare single ventricular ectopic (VE) beats with a median of 2 VE beats over the 24 h (range: 1–3 beats). All VE beats were isolated, and all dogs were free of clinical signs without medications. Nine of the 10 Doberman Pinschers with DCM had either atrial or ventricular arrhythmias at the time of sample collection. One dog had concurrent atrial fibrillation while the other eight had VE beats, six of which warranted anti-arrhythmic therapy. Four of the dogs with MMVD had concurrent occasional atrial ectopy. None of these dogs required an AECG or anti-arrhythmic therapy. All healthy non-Boxer dogs had unremarkable 24-h AECGs.

### Anti-desmoglein-2 antibody detection

**Figure 1:**
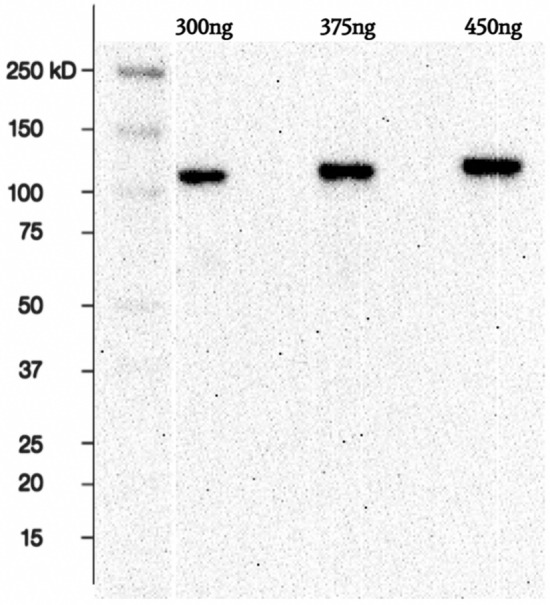
Western blot (cropped) of validation of the desmoglein-2 recombinant protein detection by commercially available anti-desmoglein-2 antibodies. Each well was loaded with increasing quantity (ng) of recombinant desmoglein-2 protein on the same gel. Original uncropped blot is displayed in Supplementary Figs. S1 and S2.

Serum autoantibodies against desmoglein-2 in the dogs were detected indirectly using immunoblot analysis. Detection of recombinant desmoglein-2 was first validated using commercially available anti-desmoglein-2 antibodies on recombinant desmoglein-2 that was denatured, reduced and separated by sodium dodecyl sulfate–polyacrylamide gel electrophoresis, which revealed clear bands at the expected molecular weight of ~ 115 kDa. Increasing loading amount of the recombinant desmoglein-2 protein resulted in greater band density and served as a positive control (Fig. 1).

**Figure 2:**
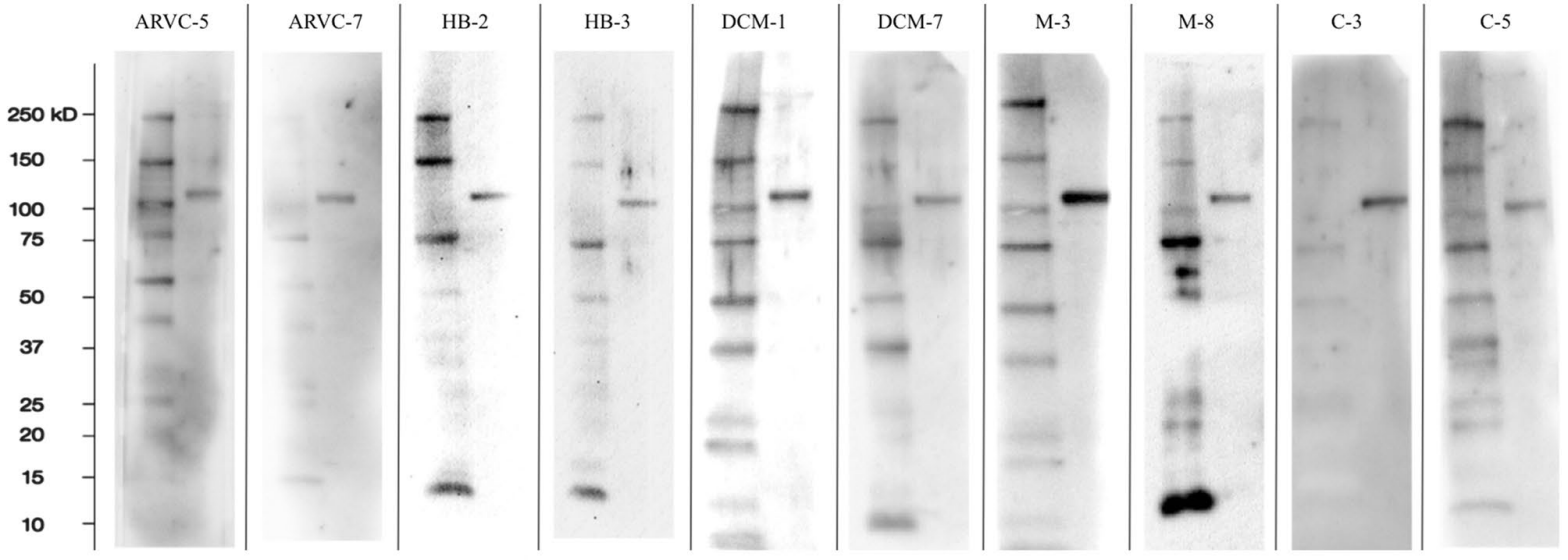
Representative Western blot images for two study dogs from each group, with study number above the blot. Each blot contains a molecular weight ladder in the well on the left and the desmoglein-2 band on the right at a molecular weight of 115 kDa. Blots were cut into two well sections, as above, from different gels before exposure with each individual canine serum. Western blots (cropped and uncropped) for all study dogs are displayed in Supplementary Figs. S3–S12.

Probing of recombinant desmoglein-2 with diluted dog serum demonstrated that autoantibodies to desmoglein-2 were present in all dogs, regardless of disease status and breed. All serum samples resulted in immunodetection of the recombinant protein at the expected molecular weight of ~ 115 kDa. Proteins bands were variable in their intensity, but clearly detectable. Figure 2 displays representative Western blot images for two dogs in each study population.

### Anti-desmoglein-2 antibody expression

**Figure 3:**
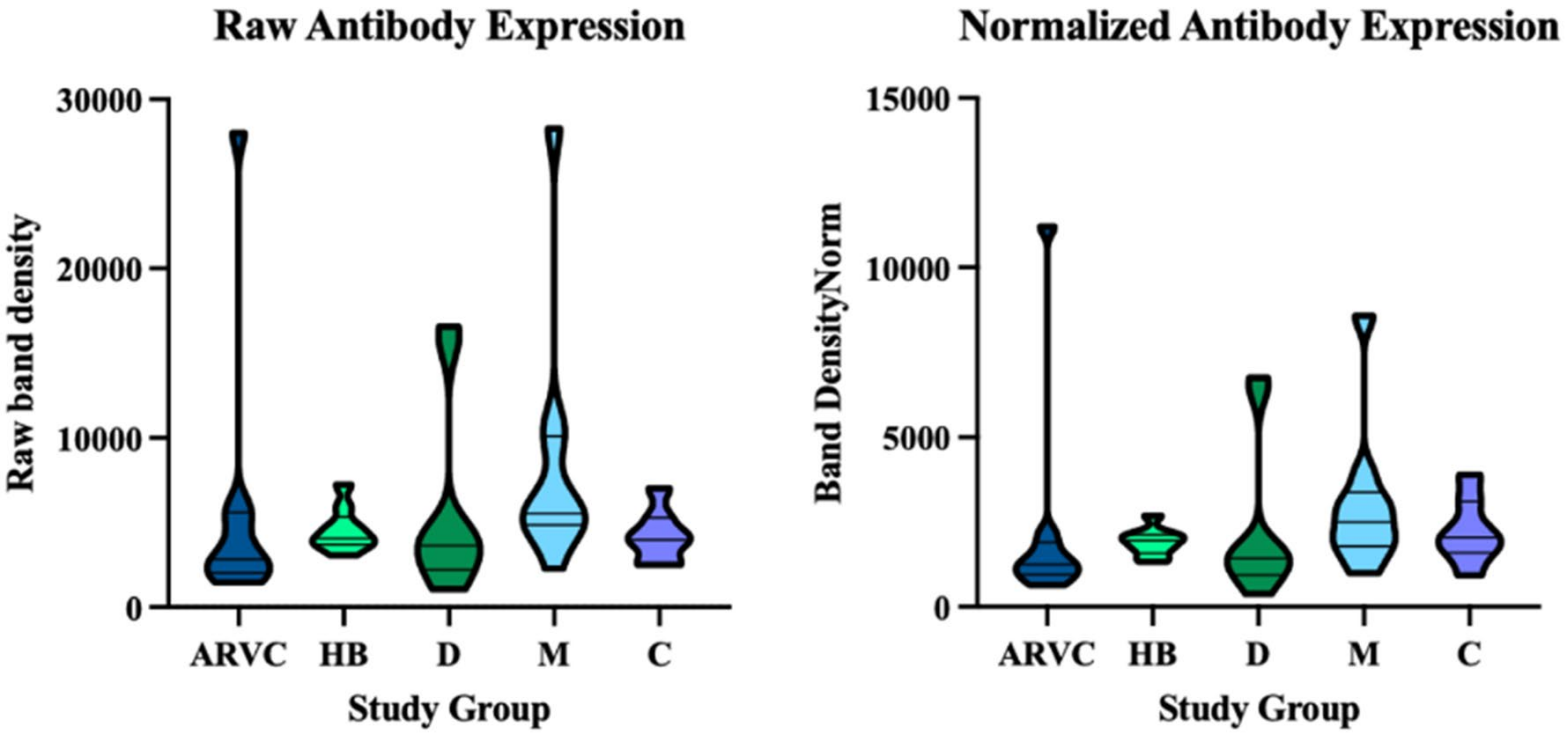
Violin plots displaying the raw and normalized anti-desmoglein-2 antibody densities by group. The width of each plot is proportionate to the number of points at that density. The top line within each plot represents the first quartile range, the middle line represents the median, and the bottom line represents the first quartile range. *ARVC* ARVC Boxer dogs, *HB* healthy Boxer dogs, *D* Doberman Pinschers with DCM, *M* small dogs with MMVD, *C* healthy non-Boxer dogs.

Serum autoantibody concentrations to desmonglein-2 was indirectly and semi-quantitatively measured by densitometry. Densitometric signal was normalized to globulin concentrations in serum. There was no significant difference in anti-desmoglein-2 antibody concentrations between any of the study groups, both when the raw densities (p = 0.0992) and normalized densities (p = 0.074) were compared. Band densities by group are displayed in Fig. 3. Raw densities weakly correlated with globulin concentration (r = 0.295, CI − 0.004–0.55; p = 0.0465) across all dogs. Autoantibody expression did not correlate significantly with body weight (raw r = 0.13, p = 0.129; normalized r = − 0.22, p = 0.143) nor with age (raw r = 0.13, p = 0.380; normalized r = − 0.025, p = 0.871).

When considering only the three groups of dogs with cardiac disease (ARVC Boxers, Doberman Pinschers with DCM, and MMVD dogs), both raw and normalized anti-desmoglein-2 antibody expression significantly correlated with left ventricular chamber size (measured as normalized left ventricular internal dimension in diastole, LVIDDn). Autontibody expression, however, did not correlate significantly with left atrial size (measured as left atrial to aortic ratio in short axis, LA:Ao). Additionally, when assessing the burden of ventricular ectopy of the ARVC Boxer group based on AECG, there was a strong significant correlation of both raw and normalized band densities with the VE complexity on each dog’s AECG at diagnosis, but not with the total VE burden. Table 3 shows the correlation coefficients and p-values for these assessments.

**Table 3:** Correlation between echocardiographic measurements and anti-desmoglein-2 antibody concentration in all dogs with cardiac disease are shown. Correlation between ventricular ectopy (VE) burden and complexity and auto-antibody expression for Boxers with ARVC are also shown.

	LVIDDn (ARVC, D, and M dogs)	LA:Ao (ARVC, D, and M dogs)	Total VE on AECG (ARVC dogs only)	VE complexity on AECG (ARVC dogs only)
Raw band density	r = 0.423	r = 0.160	r = 0.383	r = 0.841
	p = 0.020	p = 0.407	p = 0.313	p = 0.007
Normalized band density	r = 0.428	r = 0.136	r = 0.133	r = 0.719
	p = 0.018	p = 0.483	p = 0.744	p = 0.037

## Discussion

This study is the first to evaluate dogs of various breeds and cardiac diseases for the presence of autoantibodies to the desmosomal protein, desmoglein-2. Our results indicate that anti-desmoglein-2 antibodies are present in all dogs, regardless of breed or cardiac disease. Specifically, antibodies were detected in Boxers with ARVC, healthy Boxers, Doberman Pinschers with DCM, various small breeds of dogs with MMVD, and various healthy breeds of dogs. There was no correlation found between the expression of anti-desmoglein-2 antibodies and study dog age or body weight. Furthermore, no significant difference between the study disease groups and antibody expression was found. These findings indicate that anti-desmoglein-2 antibodies are unlikely to have utility as a diagnostic test for ARVC in Boxers as they are not specific to the disease and were repeatedly found in healthy dogs.

Our findings differ from those of two previous studies assessing autoimmunity in human ARVC, one of which (Chatterjee et al.) specifically evaluated both human patients and Boxer dogs for the presence of anti-desmoglein-2 antibodies^14,15^. While our results indicate anti-desmoglein-2 antibodies to be present in Boxers with ARVC, we also found them to be present consistently in healthy Boxers and healthy non-Boxers. This is in contrast with both studies by Chatterjee et al. and Caforio et al., which found significantly more frequent and greater concentrations of autoantibodies in people affected with ARVC compared to healthy controls. A more recent study assessing healthy human athletes with physiologic right ventricular enlargement found that anti-desmoglein-2 antibodies were not present, indicating further that they may play pathologic role^22^. However, Caforio et al. also assessed heathy relatives of people with ARVC and patients with noninflammatory and ischemic cardiac disease as well as healthy unrelated blood donors, finding that anti-heart and anti-intercalated disc antibodies were present in some of these patients, albeit at a lower frequency compared to patients with ARVC and affected relatives. Additionally, while Chaterjee et al. found autoantibodies to be discriminatory for ARVC in the Boxer dog there are important differences between their methods and the present study. Specifically, sera in their study were not protease-inhibited and importantly they did not alter the secondary antibodies used for dog sera, continuing to use goat-anti-human secondary antibodies to probe blots rather than canine specific antibodies. Furthermore, from where dog sera were obtained and for how long it was stored prior to analysis was not stated. Finally, little information was provided regarding characteristics and clinic findings for Boxers with ARVC and no information was provided regarding their subset of healthy Boxers and mixed breed dogs. Herein we report that anti-desmolgein-2 antibodies were found in all dogs evaluated without a significant difference in antibody expression between any of the study groups. Our findings are in alignment with those of a recently presented abstract assessing Boxers with ARVC and healthy Boxers for anti-desmoglein-2 antibodies and indicate that further research is required in this field^23^.

Similar to prior studies, a significant correlation between autoantibody concentrations and certain markers of disease severity was found in this study. Our results indicate that increasing concentrations of anti-desmoglein-2 antibodies correlate with the degree of left ventricular dilatation in dogs with structural cardiac disease, regardless of whether that disease is ARVC, DCM, or MMVD. Within the studied group of Boxers with ARVC, we also found a significant correlation between autoantibody expression and the complexity of ventricular arrhythmias, a marker of a more severe disease state. These results may indicate that anti-desmoglein-2 antibodies are a sequelae of cardiomyocyte damage and thus in a more severe disease state autoantibody concentration is higher. Alternatively, if playing a role in pathogenesis of disease, autoantibody concentrations being higher may result in a worsened disease state. Our finding of anti-desmoglein-2 antibodies consistently in both healthy Boxers and healthy non-Boxer dogs would support the former hypothesis, however longitudinal studies with sequential measurements of antibody expression and markers of disease severity in the same individual would be needed to elucidate this.

There is significant evidence that myocardial inflammation and an activated immune system play a role in ARVC, but it remains unclear whether this inflammation is an inciting insult, a reaction to cardiomyocyte destruction, or both^6,16,24^. Multiple small animal models of ARVC implicate inflammation in the pathogenesis of disease^25–27^. Inflammatory cells including T-cell lymphocytes, macrophages, neutrophils, and mast cells are commonly found in the myocardium of people with ARVC, especially in regions affected by fibro-fatty change^24^. Higher concentrations of circulating inflammatory cytokines and C-reactive protein have been found in patients with ARVC and associated with arrhythmic events^28,29^. Myocardial lymphocytic infiltration has also been shown in Boxers with ARVC and such inflammation was significantly associated with dogs that experienced sudden cardiac death.^4^ In humans, such myocardial inflammation is thought to be associated with the so called “hot phase” of disease in which patients are often initially misdiagnosed with myocarditis and may be associated with more severe arrhythmias and a greater risk of sudden cardiac death^6,30^. Again, it remains unclear whether this inflammation results in cardiomyocyte death and progression of disease or is the immune system’s reaction to cardiomyocyte destruction.

We hypothesize that autoantibodies to desmoglein-2 are a sequelae of cardiomyocyte death during which previously hidden or infrequently exposed epitopes are released and stimulate an immune reaction. Our results indicate that this is not a process specific to ARVC or even cardiac pathology, as healthy dogs and dogs with DCM and MMVD also possessed these antibodies. In fact, a recent study identified a significant increase in anti-desmoglein-2 antibodies in people recently infected with COVID-19 compared to both a control group and patients with ARVC^31^. It was an unexpected finding that all healthy dogs in this study had some level of anti-desmoglein-2 antibodies, suggesting that some degree of antigenic stimulation to desmosomal proteins is normal in dogs. Despite their immense importance in structural integrity and cell function, the desmosome remains a dynamic structure with frequent turnover of desmosomal proteins in health^32^. Our results indicate a weak correlation between autoantibody concentration and left ventricular dilatation and a strong correlation between autoantibody concentrations and the complexity of VE. These findings together suggest that autoantibody concentrations increase with increasing severity of disease. It is possible that such elevations correlate with the “hot phase” of ARVC in Boxer dogs and could signal a worsening of arrhythmias. While we did not find a significant correlation between total VE beats and autoantibody expression, many of the Boxers in our study group were already on anti-arrhythmic treatment and possibly in a better controlled or less inflammatory stage of disease making this difficult to assess. A longitudinal study would be able to better assess how autoantibody concentrations change with improvement or worsening of ventricular arrhythmias.

Limitations of this study include the small number of dogs in each study group. This could have resulted in type II error and therefore it is possible that with a larger study population, a difference in autoantibody expression between study groups would be found. Furthermore, while the healthy Boxers were thoroughly screened for any cardiac disease, it is possible that some of the dogs included could develop ARVC later in life or even already harbored an occult disease state on a molecular or cellular level. Also, dogs included in the ARVC, DCM, and MMVD groups were recruited from the clinic and therefore the diagnostics performed and treatments for these dogs were heterogenous and at the discretion of the attending clinician. In particular, the point at which antiarrhythmic therapy was instituted and the drug chosen was clinician dependent. Additionally, AECGs and echocardiograms were performed and analyzed by multiple individuals which may have introduced some variability. One dog within the ARVC group (ARVC-5) did not have an AECG performed contemporaneously with serum collection, which could have impacted the accuracy of a correlation between ectopy and autoantibody expression in this dog. Type III ARVC is difficult to diagnose, as other phenocopies can present similarly. It is possible that the two Boxers with type III ARVC included in this study had another etiology for their DCM phenotype. As our study results show that anti-desmoglein-2 antibodies are not disease specific, even if misclassified, this would not have significantly affected our overall conclusions. Dogs were also enrolled at various stages of their respective diseases, whether at first diagnosis or a recheck visit, and it is possible that autoantibody expression changes with progression of disease as highlighted in our discussion. Again, longitudinal studies would be helpful to determine how anti-desmoglein-2 antibody levels change within the individual and how these change correlate with disease progression. Detection of autoantibodies to our target protein was indirectly measured using Western blot, which is semiquantitative in nature and subjected to many analytic variabilities. Other more quantitative and repeatable methods like ELISA or immunosensors may yield more consistent results but are not commercially available for use in dogs. Lastly, while the measurement of band densities was performed in a blinded manner, all other analyses were not and therefore could have introduced unintended bias.

In conclusion, we report the first veterinary study assessing dogs for autoantibodies to desmoglein-2. Our findings indicate these autoantibodies are present both in cardiac disease and health across a range of canine breeds. Anti-desmoglein-2 antibodies were found in all dogs evaluated, including Boxers with ARVC, Doberman Pinchers with DCM, small breed dogs with MMVD, and both healthy Boxers and non-Boxer breeds. There was a weak correlation between autoantibody expression and the degree of left ventricular dilatation in dogs with cardiac disease and a strong correlation with the complexity of ventricular arrhythmias in Boxers with ARVC. Together, this suggests that autoantibody concentrations coincide with the severity of disease and may serve as a biomarker for worsening ventricular remodeling or arrhythmias, however larger studies are needed to better assess this.

## Materials and methods

### Animals

This study was approved by the Institutional Animal Care and Use Committee (IACUC: 21984) at the University of California, Davis. Methods were carried out in accordance with university guidelines, the approved IACUC committee protocol, and the ARRIVE guidelines and regulations. Animals used in this study were client-owned dogs recruited to the University of California, Davis Veterinary Medical Teaching Hospital. Dogs were recruited for and prospectively enrolled in the study between November 2020 and July 2022 and comprised five different groups: (1) Boxer dogs with a diagnosis of ARVC; (2) healthy Boxer (HB) dogs; (3) Doberman Pinschers with a diagnosis of DCM; (4) small breed dogs (< 15 kg) with a diagnosis of MMVD, and (5) healthy non-Boxer dogs. All owners were informed of the study requirements and written consent was obtained for each study participant. All Boxer dogs were genotyped for the striatin gene mutation. Doberman Pinschers were genotyped for the DCM1 (PDK4 gene) and DCM2 (titin gene) mutations. All genetic testing was performed by the North Carolina State University Veterinary Cardiac Genetics Laboratory.

A diagnosis of ARVC in Boxers was made by the attending clinician for each case based on a combination of clinical signs, genotype, ECG findings, echocardiography, and 24-h ambulatory ECG (AECG) results. Inclusion criteria for the ARVC Boxer group were purebred Boxer breed, a positive striatin mutation status (heterozygous or homozygous), and at least one of the following: (1) > 300 VPCs on 24-h AECG, (2) malignant ventricular arrhythmias warranting anti-arrhythmic therapy prior to AECG, and/or (3) a dilated and poorly contractile left ventricle on echocardiogram. Therefore, dogs could be classified as having type II ARVC (solely arrhythmogenic) or type III ARVC (dilated cardiomyopathy phenotype + /− arrhythmias). Dogs were required to have had at least one ECG and echocardiogram to be in included. At the studied university it is standard of care to perform AECG prior to anti-arrhythmic therapy unless in-hospital ECG shows runs of ventricular tachycardia at a heart rate over 180 bpm, R on T phenomenon, and/or the dog was syncopal. Dogs were excluded if they had any other hemodynamically significant echocardiographic diagnosis (including congenital or acquired diseases and cardiac masses) or if systemic work-up revealed another possible etiology for ventricular arrhythmias or a dilated cardiomyopathy phenotype.

Clinically healthy Boxers were actively recruited from hospital clientele and staff. Inclusion criteria were a negative striatin mutation, normal echocardiogram, normal five minute-surface ECG and < 100 single VPCs on 24-h AECG. All healthy Boxer echocardiograms, ECGs, and AECG analyses were performed by a single investigator (ALW). Doberman Pinschers were diagnosed with dilated cardiomyopathy, with or without secondary congestive heart failure, by the attending clinician for each case. Diagnosis of DCM was made based on echocardiogram, ECG, and AECG results in accordance with the current European Society of Veterinary Cardiology guidelines^33^. Dogs weighing less than 15 kg were enrolled into the MMVD group if they had a diagnosis of MMVD and were classified according to the ACVIM consensus statement as stage B2 or C based on echocardiography by the attending clinician^34^. For both the DCM and MMVD groups, a diagnosis of secondary congestive heart failure was made based on the presence of pulmonary edema on thoracic radiographs and concurrent clinical signs (tachypnea, dyspnea, and/or cough). When present, the type and severity of arrhythmias were recorded for each case in the DCM and MMVD groups.

Clinically healthy dogs of various non-Boxer breeds were recruited into the study from hospital clientele and staff. Dogs of any age and breed were eligible. All dogs underwent a complete echocardiogram, 5-min ECG, and 24-h AECG. Any echocardiographic diagnosis other than trivial valvular regurgitation resulted in exclusion. Both the ECGs and AECGs were required to be normal. Echocardiograms, ECGs and AECGs for this group were performed by a single investigator (ALG).

All echocardiograms in the study were performed by either a board-certified veterinary cardiologist or a veterinary cardiology resident under direct supervision of a board-certified veterinary cardiologist. The echocardiographic images were reviewed by a single investigator (ALW) for all dogs included in the study. Dogs were excluded from any group if they were known to have a neoplastic, immune-mediated, or infectious disease process. Dogs receiving systemic corticosteroids or other immunosuppressive drugs were also excluded.

### Holter data acquisition and processing

All dogs within the ARVC group that survived to discharge had at least one 24-h AECG performed. Data from prior AECGs, if performed, was also collected and reviewed by a single investigator (ALW). All healthy Boxers and healthy non-Boxer dogs had a 24-h screening AECG placed as part of the study. AECGs were not analyzed for Dobermans with DCM or dogs within the MMVD group. AECGs were performed contemporaneously with serum sampling in all but one dog; one dog in the ARVC Boxer group (ARVC-5) only had previous AECG results from 14 months and 3 years prior. All AECGs were analyzed prospectively using either Vision 5 (Mortara Instrument, Inc.) or Trillium (Forest Medical, LLC) software systems. The software automatically annotates normal and abnormal complexes; however, all recordings were visually inspected, and all mis-labeled beats were corrected in order to accurately determine the frequency and complexity of ectopy if present. Portions of the recordings with motion-related artifact that was significant enough to preclude accurate interpretation was labeled as artifact and not quantified for analysis. The total numbers of supraventricular and ventricular ectopic beats were recorded. Additionally, supraventricular and ventricular arrhythmias were classified based on a complexity scale: 0 = no arrhythmias present, 1 = only single premature complexes, 2 = couplets, 3 = triplets, or 4 =  ≥ 4 consecutive ectopic beats^35,36^. Ventricular arrhythmias were classified based on the instantaneous heart rate (HR) as premature (HR ≥ 160), accelerated idioventricular (HR = 100–159), or escape (HR ≤ 99 bpm) complexes. This complexity scale for ectopy was also applied to the AECG results of the Boxers with ARVC that had AECGs performed and analyzed off-site.

### Sample collection and handling

A single blood draw was performed for each subject. Non-additive collection tubes were used, and the blood allowed to clot then immediately centrifuged at 3000 rpm (1734 G) for 15 min to separate serum. Serum was aliquoted into 500 ul amounts with 5 ul of a commercial protease inhibitor added and placed into cryovials for storage. All samples were stored at − 80 degrees Celsius until used for western blot analysis. Globulin concentrations were obtained for each study dog also using serum. Measurement of all globulin concentrations was performed at the UC Davis Veterinary Medical Teaching Hospital clinical diagnostic laboratory (Roche cobas c501 system).

### Western blot analysis

Recombinant human desmoglein-2 protein (DSG2-1601H, Creative Biomart, Shirley, NY) was reconstituted per manufacturer’s instructions and diluted to a concentration of 100 ng/ul with the addition of 1X protease inhibitor (Thermo Fischer Scientific Halt protease inhibitor). The reconstituted protein was then aliquoted and stored at – 80 °C in cryovials for further analysis.

Initial validation of the protein detection was performed using a rabbit monoclonal anti-desmoglein-2 antibodies (DSG2 antibody, Abcam, Waltham, MA) followed by horseradish peroxidase-conjugated goat anti-rabbit IgG (Abcam, Waltham, MA) as secondary antibodies. Following validation of the recombinant desmoglein-2 protein, optimization of protein detection was performed using various concentrations of primary and secondary antibodies and quantities of recombinant protein. Based on the results of these optimization blots, the protocol for western blot analysis of study samples was developed.

All study samples were analyzed using a standardized protocol in a single laboratory. Recombinant desmoglein-2 proteins (Bio-Rad, Hercules, CA) were first denatured and reduced by boiling in 1 × Laemmli buffer with 2-mercaptoethanol. A total of 375 ng of recombinant desmoglein-2 protein was loaded into each well of precast 4–20% polyacrylamide gels (Mini-protean TGX gels, BioRad, Hercules, CA), alternating with loading of a protein ladder (BioRad, Hercules, CA) and separated by SDS-PAGE before transferring to polyvinylidene difluoride membrane (Immun-blot, BioRad, Hercules, CA) at 60 V for 70 min. Adequate protein transfer was confirmed via Ponceau S solution (Sigma Aldrich, St. Louis, MO). Blots were then blocked with 10% bovine serum albumin (BSA) in Tris buffered saline with 0.05% Tween (TBST) (overnight, 4 °C). Blots were then cropped into sections so that each lane containing the recombinant protein was paired with a protein ladder. Canine sera were thawed at room temperature and diluted 1:100 in TBST with 5% BSA. The cut blots were then incubated in sera from a single study individual for two hours at room temperature, followed by washing in and secondary body incubation with rabbit anti-dog secondary antibody (1:20,000, Abcam, 136759) for one hour at room temperature. After washing, immunoreactive bands were detected using an enhanced chemiluminescence kit (Prometheus ProSignal Dura, Genesee Scientific, El Cajon, CA) and imaged using commercially available imaging system (ProteinSimple FluorChem E system) with an exposure of 60 s. Detected bands were then quantified via densitometry using the ImageJ software correcting for the background density of each blot by a single investigator in a blinded manner.

### Statistical analysis

Band densities are presented both as the raw measured density and as a normalized value. Normalization of densitometry was calculated using each dog’s measured globulin concentration and using the formula:

$${\text{Density}}_{{{\text{normalized}}}} {\text{ = raw density/globulin concentration }}\left( {\text{g/dL}} \right)$$

Study dog characteristics (body weight, age, globulin concentration) and the raw and normalized densities were tested for normality via D’Agostino and Pearson test. Normally distributed data and nonparametric data are displayed as mean (+ /− SD) and median [IQR], respectively. Differences between groups for numerical discrete data were tested for via a one-way ANOVA for normally distributed data or a Kruskal–Wallis test for nonparametric data. Correlation testing was performed for both raw and normalized densities using a Spearman test and the correlation coefficient and p-value are reported. Significance was considered to be a p-value < 0.05.

## Supplementary Information

Supplementary Figures.

## Data Availability

The datasets used and/or analyzed during the current study are available from the corresponding author on reasonable request.

## References

[1] Meurs KM (2017). Arrhythmogenic right ventricular cardiomyopathy in the boxer dog: An update. Vet. Clin. North Am. Small Anim. Pract..

[2] Meurs KM (2014). Natural history of arrhythmogenic right ventricular cardiomyopathy in the boxer dog: A prospective study. J. Vet. Intern. Med..

[3] Meurs KM (2013). Association of dilated cardiomyopathy with the striatin mutation genotype in boxer dogs. J. Vet. Intern. Med..

[4] Basso C (2004). Arrhythmogenic right ventricular cardiomyopathy causing sudden cardiac death in boxer dogs: A new animal model of human disease. Circulation.

[5] Vischer AS (2017). Arrhythmogenic right ventricular cardiomyopathy in Boxer dogs: The diagnosis as a link to the human disease. Acta Myol..

[6] Cicenia M, Drago F (2022). Arrhythmogenic cardiomyopathy: Diagnosis, evolution, risk stratification and pediatric population—Where are we?. J. Cardiovasc. Dev. Dis..

[7] Oxford EM (2007). Molecular composition of the intercalated disc in a spontaneous canine animal model of arrhythmogenic right ventricular dysplasia/cardiomyopathy. Heart Rhythm.

[8] Oxford EM (2011). Ultrastructural changes in cardiac myocytes from Boxer dogs with arrhythmogenic right ventricular cardiomyopathy. J. Vet. Cardiol..

[9] Meurs KM (2010). Genome-wide association identifies a deletion in the 3’ untranslated region of striatin in a canine model of arrhythmogenic right ventricular cardiomyopathy. Hum. Genet..

[10] Cattanach BM, Dukes-McEwan J, Wotton PR, Stephenson HM, Hamilton RM (2015). A pedigree-based genetic appraisal of Boxer ARVC and the role of the Striatin mutation. Vet. Rec..

[11] Ohno S (2016). The genetic background of arrhythmogenic right ventricular cardiomyopathy. J. Arrhythm..

[12] Mattesi G, Zorzi A, Corrado D, Cipriani A (2020). Natural history of arrhythmogenic cardiomyopathy. J. Clin. Med..

[13] Paul M, Schulze-Bahr E (2020). Arrhythmogenic right ventricular cardiomyopathy: Evolving from unique clinical features to a complex pathophysiological concept. Herz.

[14] Chatterjee D (2018). An autoantibody identifies arrhythmogenic right ventricular cardiomyopathy and participates in its pathogenesis. Eur. Heart J..

[15] Caforio ALP (2020). Evidence from family studies for autoimmunity in arrhythmogenic right ventricular cardiomyopathy. Circulation.

[16] Meraviglia V, Alcalde M, Campuzano O, Bellin M (2021). Inflammation in the pathogenesis of arrhythmogenic cardiomyopathy: Secondary event or active driver?. Front. Cardiovasc. Med..

[17] Nikolaev VO (2007). A novel fluorescence method for the rapid detection of functional beta1-adrenergic receptor autoantibodies in heart failure. J. Am. Coll. Cardiol..

[18] Wallukat G, Nissen E, Morwinski R, Müller J (2000). Autoantibodies against the beta- and muscarinic receptors in cardiomyopathy. Herz.

[19] Wess G (2019). Doberman pinschers present autoimmunity associated with functional autoantibodies: A model to study the autoimmune background of human dilated cardiomyopathy. PLoS ONE.

[20] Jahns, R. *et al. Autoantibodies Activating Human 1-Adrenergic Receptors Are Associated With Reduced Cardiac Function in Chronic Heart Failure*. http://www.circulationaha.org (1999). 10.1161/01.cir.99.5.649 9950662

[21] Becker N-P, Goettel P, Mueller J, Wallukat G, Schimke I (2019). Functional autoantibody diseases: Basics and treatment related to cardiomyopathies. Front. Biosci. (Landm. Ed.).

[22] Dorian D (2021). A novel arrhythmogenic right ventricular cardiomyopathy (ARVC) biomarker—Anti-DSG2—Is absent in athletes with right ventricular enlargement. CJC Open.

[23] dos Santos, L., Cunningham, S., Hamilton, R. & Fatah, M. Anti-desmosomal Antibody as a Potential Biomarker of Arrhythmia Burden in Boxers with Arrhythmogenic Ventricular Cardiomyopathy. *2022 ACVIM Forum Research Abstract Program* Preprint at (2022).

[24] Campuzano O (2012). Arrhythmogenic right ventricular cardiomyopathy: Severe structural alterations are associated with inflammation. J. Clin. Pathol..

[25] Lubos N (2020). Inflammation shapes pathogenesis of murine arrhythmogenic cardiomyopathy. Basic Res. Cardiol..

[26] Asimaki A (2014). Identification of a new modulator of the intercalated disc in a zebrafish model of arrhythmogenic cardiomyopathy. Sci. Transl. Med..

[27] Chelko SP (2019). Therapeutic modulation of the immune response in arrhythmogenic cardiomyopathy. Circulation.

[28] Asimaki A (2011). Altered desmosomal proteins in granulomatous myocarditis and potential pathogenic links to arrhythmogenic right ventricular cardiomyopathy. Circulation.

[29] Bonny A (2010). C-reactive protein in arrhythmogenic right ventricular dysplasia/cardiomyopathy and relationship with ventricular tachycardia. Cardiol. Res. Pract..

[30] Bariani R (2022). Myocarditis-like episodes in patients with arrhythmogenic cardiomyopathy: A systematic review on the so-called hot-phase of the disease. Biomolecules.

[31] Lee ECY (2022). High frequency of anti-DSG 2 antibodies in post COVID-19 serum samples. J. Mol. Cell Cardiol..

[32] Windoffer R, Borchert-Stuhlträger M, Leube RE (2002). Desmosomes: interconnected calcium-dependent structures of remarkable stability with significant integral membrane protein turnover. J. Cell Sci..

[33] Wess G, Domenech O, Dukes-McEwan J, Häggström J, Gordon S (2017). European society of veterinary cardiology screening guidelines for dilated cardiomyopathy in Doberman pinschers. J. Vet. Cardiol..

[34] Keene BW (2019). ACVIM consensus guidelines for the diagnosis and treatment of myxomatous mitral valve disease in dogs. J. Vet. Intern. Med..

[35] Ueda Y (2019). Heart rate and heart rate variability of rhesus macaques (Macaca mulatta) affected by left ventricular hypertrophy. Front. Vet. Sci..

[36] Walker AL, Ueda Y, Crofton AE, Harris SP, Stern JA (2022). Ambulatory electrocardiography, heart rate variability, and pharmacologic stress testing in cats with subclinical hypertrophic cardiomyopathy. Sci. Rep..

