# Supplementary material for: Evaluation of autoantibodies to desmoglein-2 in dogs with and without cardiac disease
Source: Sci Rep. 2023 Mar 28;13:5044. doi: 10.1038/s41598-023-32081-x (PMC10043840; doi:10.1038/s41598-023-32081-x)
Supplement: Supplementary file 1 — Supplementary Figures. [file 41598_2023_32081_MOESM1_ESM.docx]

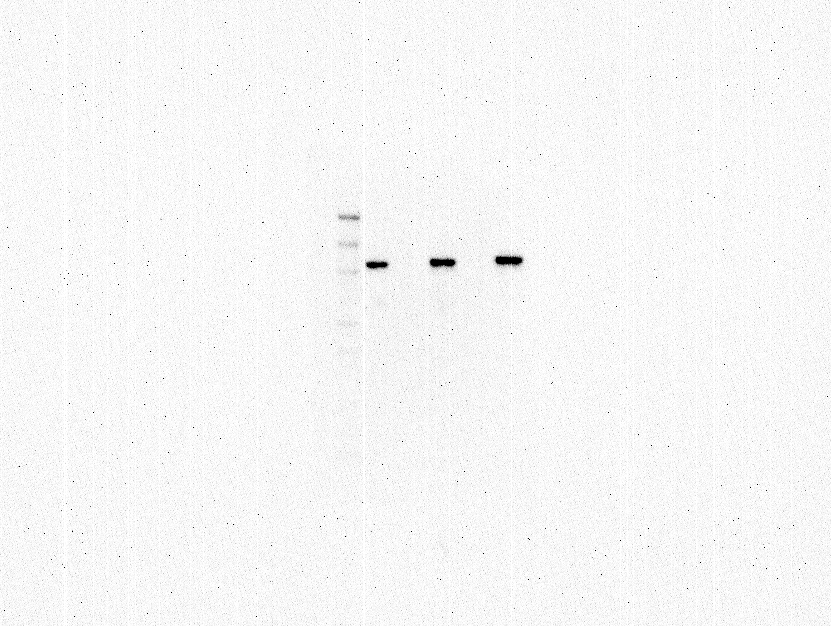


**Figure S1. Uncropped positive control Western blot image**

**
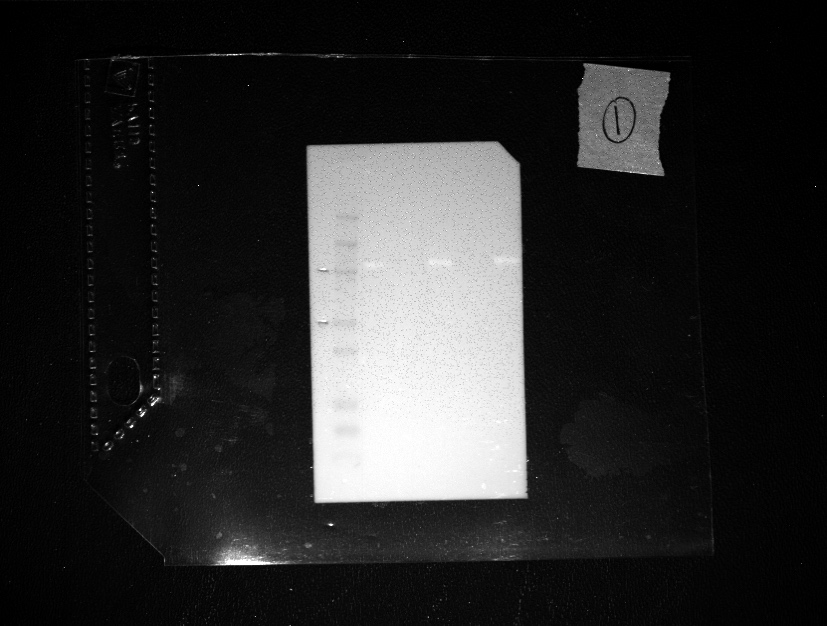
**

**Figure S2. Uncropped positive control Western blot image Epi white exposure**

**
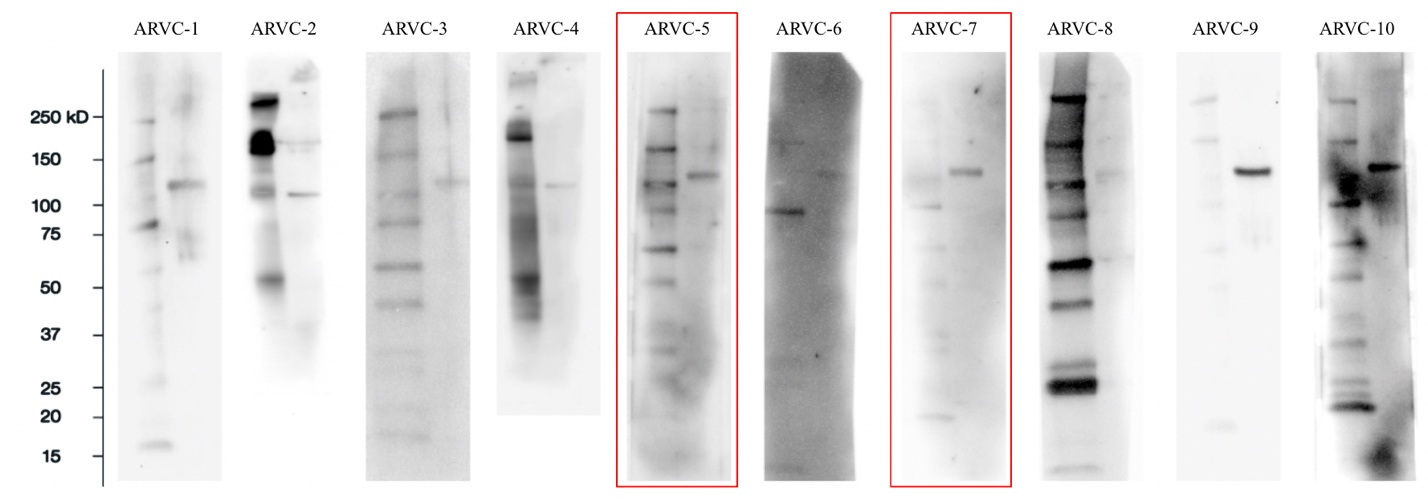
**

**Figure S3. Cropped Western blots for all ARVC dogs.** Those images displayed as representative blots in the manuscript are highlighted by a red box.


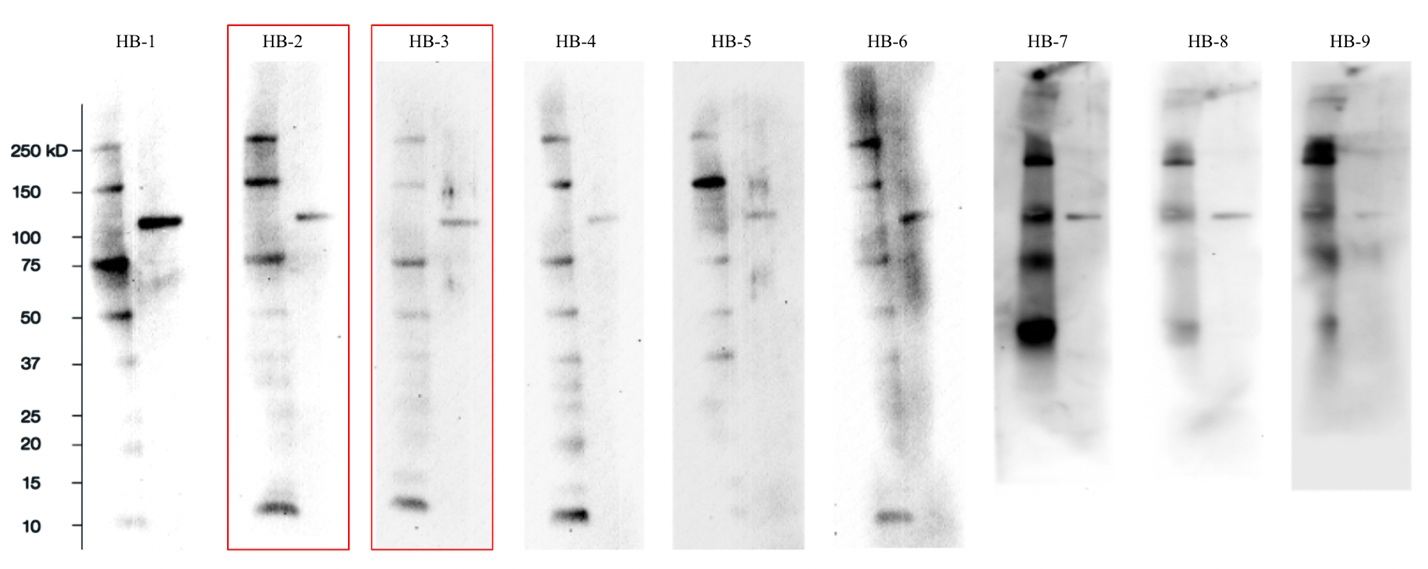


**Figure S4. Cropped Western blots for all healthy Boxer dogs.** Those images displayed as representative blots in the manuscript are highlighted by a red box.


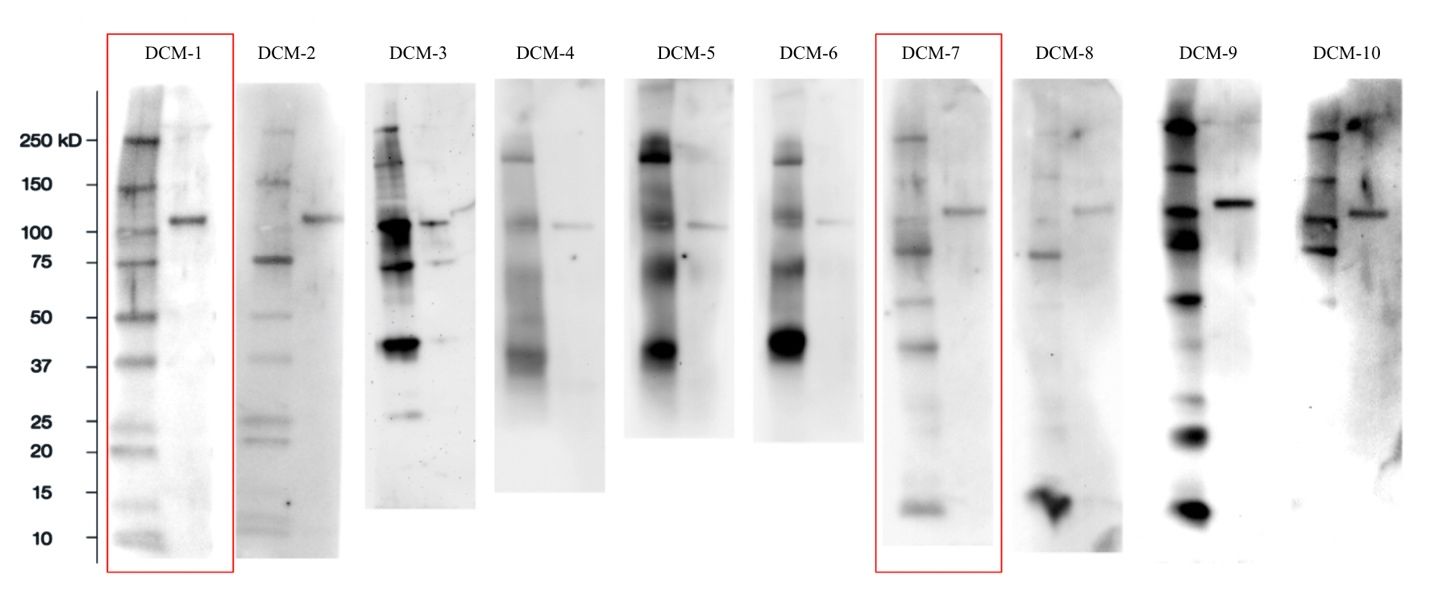


**Figure S5. Cropped Western blots for all DCM Dobermans.** Those images displayed as representative blots in the manuscript are highlighted by a red box.


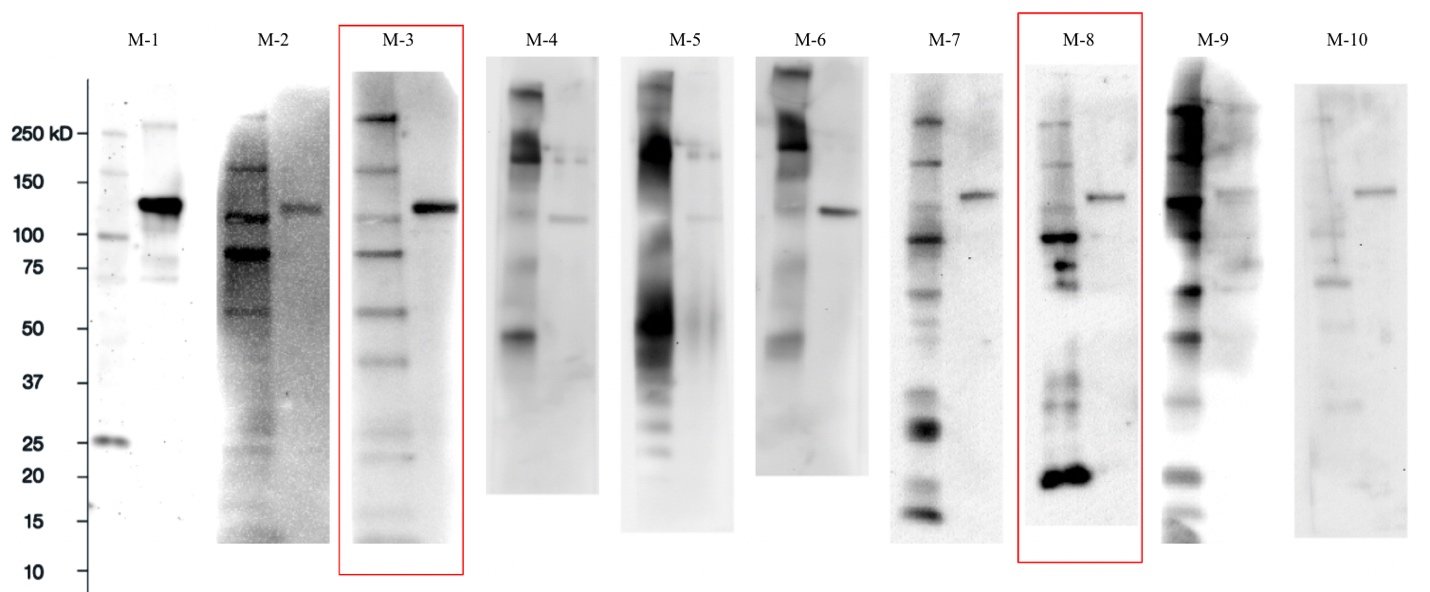


**Figure S6. Cropped Western blots for all MMVD dogs.** Those images displayed as representative blots in the manuscript are highlighted by a red box.


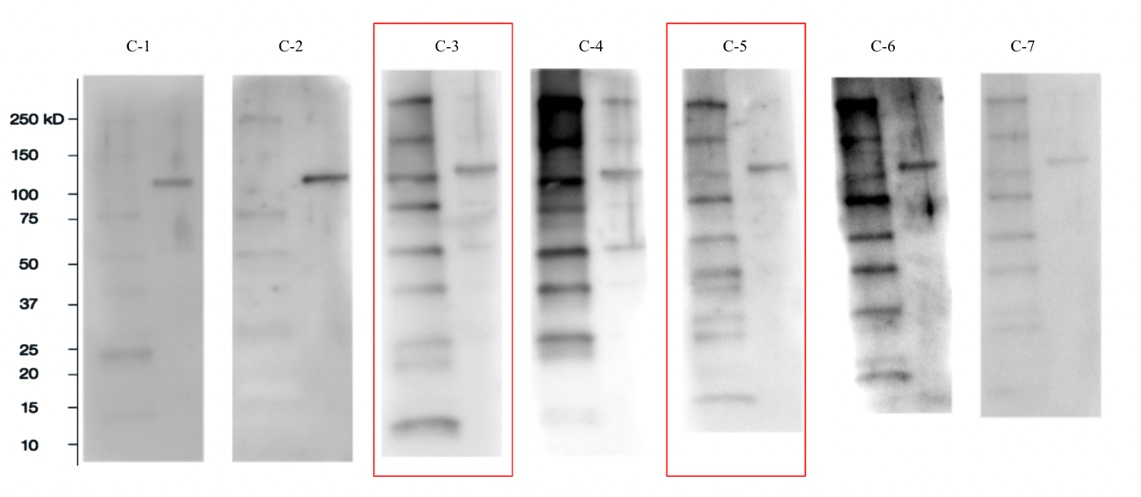


**Figure S7. Cropped Western blots for all healthy non-Boxer dogs.** Those images displayed as representative blots in the manuscript are highlighted by a red box.


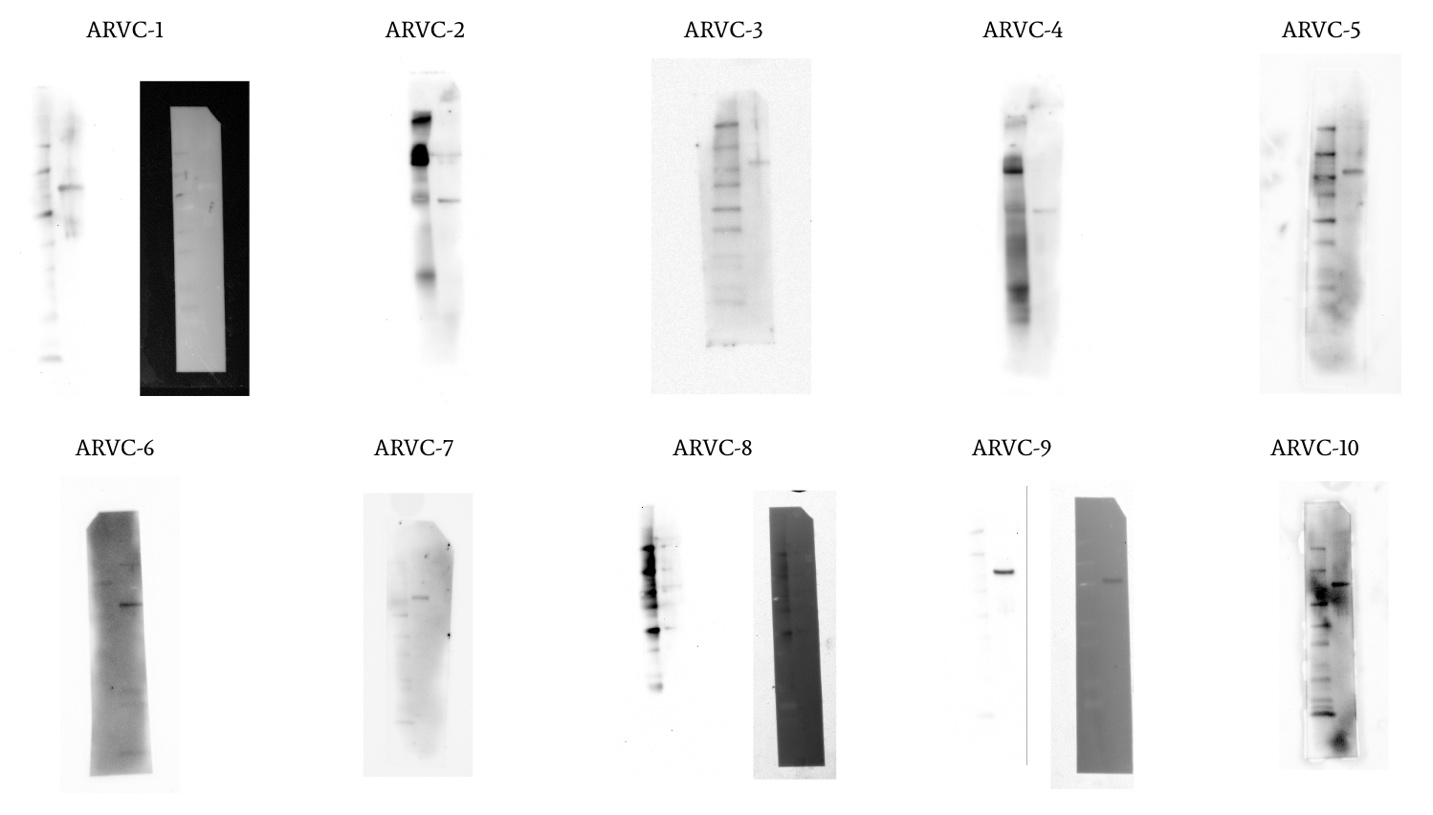


**Figure S8. Uncropped Western blots for all ARVC dogs.** For blots in which membrane edges are not clearly visible, Epi white exposures have also been included.


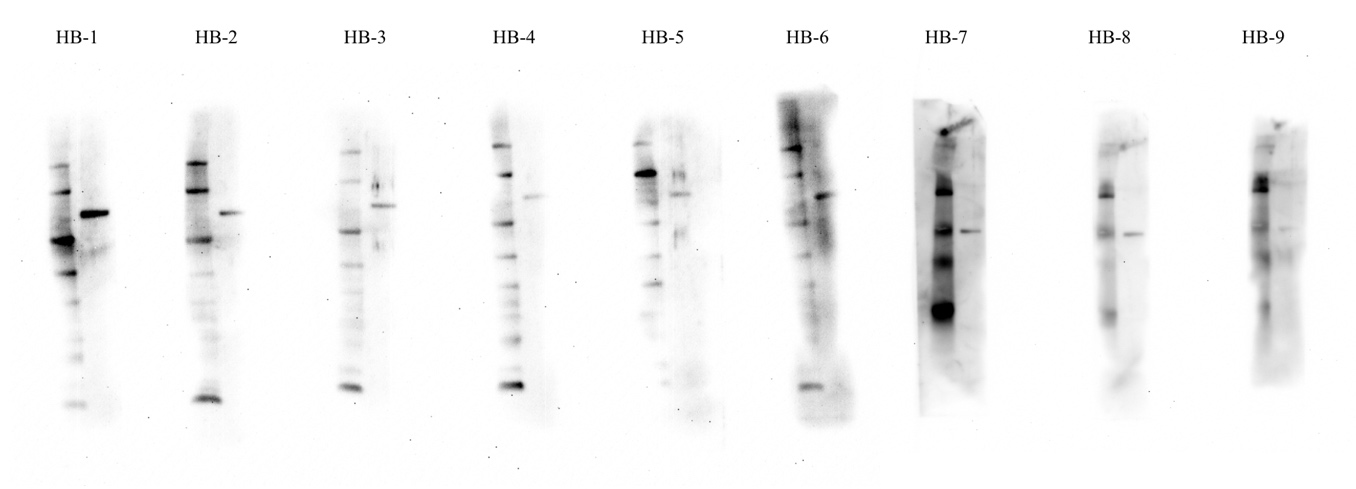


**Figure S9. Uncropped Western blots for all healthy Boxer dogs.**


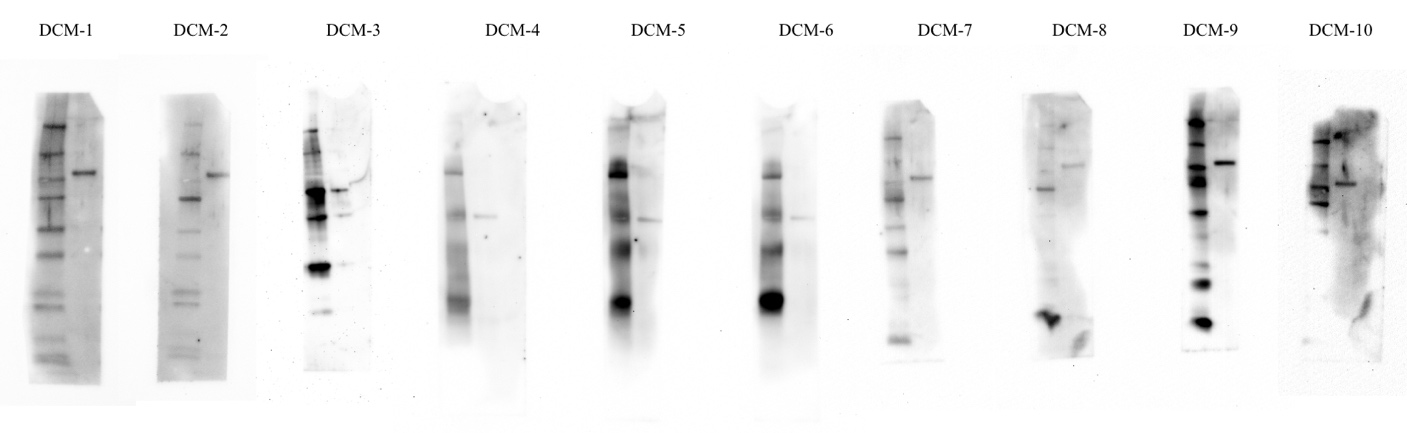


**Figure S10. Uncropped Western blots for all DCM Doberman dogs.**


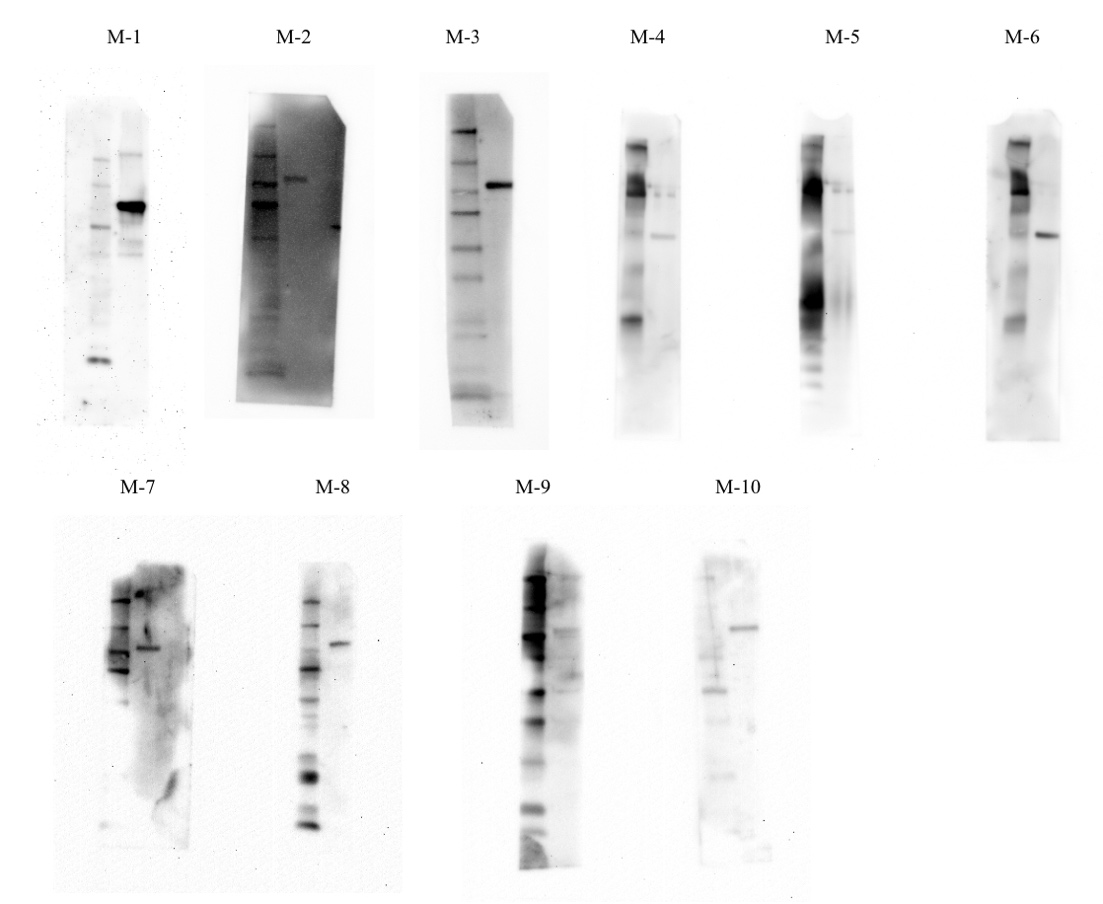


**Figure S11. Uncropped Western blots for all MMVD dogs.**


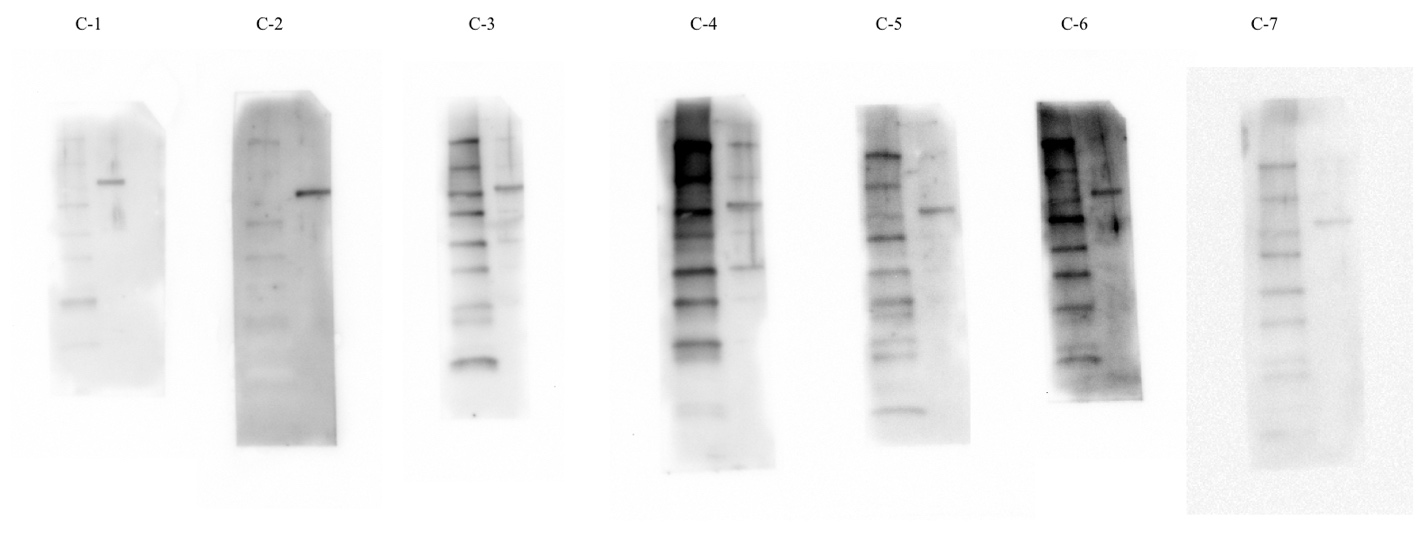


**Figure S12. Uncropped Western blots for all healthy non-Boxer dogs.**
